# Titanium-coated PEEK versus 3D-printed porous titanium cages in transforaminal lumbar interbody fusion: A meta-analysis

**DOI:** 10.1016/j.jor.2026.05.013

**Published:** 2026-05-19

**Authors:** Youngmin Yu, Thomas Cho, Jiayong Liu

**Affiliations:** Department of Orthopaedic Surgery, University of Toledo Medical Center, Toledo, OH, 43614, USA

**Keywords:** Transforaminal lumbar interbody fusion (TLIF), Polyetheretherketone (PEEK), Titanium (Ti), Titanium-coated PEEK (Ti-PEEK), Degenerative spinal disease

## Abstract

**Background:**

Transforaminal lumbar interbody fusion (TLIF) is a common procedure for degenerative spinal conditions. While polyetheretherketone (PEEK) and titanium (Ti) are established cage materials, titanium-coated PEEK (Ti-PEEK) was developed to combine PEEK's favorable modulus with Ti's osteoconductive surface. This meta-analysis compares the radiographic and clinical outcomes of modern 3D-printed porous titanium (3D-Ti) and Ti-PEEK cages in TLIF.

**Methods:**

A systematic search of PubMed, Embase, and Google Scholar was conducted up to April 2025 following PRISMA guidelines. Studies comparing 3D-Ti and/or Ti-PEEK cages in TLIF, reporting outcomes of fusion, subsidence, reoperation, or visual analogue scale (VAS) scores with a minimum 6-month follow-up were included. Given the scarcity of direct comparisons, an indirect comparison was performed using single-arm random-effects meta-analyses for each material, followed by meta-regression to estimate differences. Risk of bias was assessed using the Cochrane Risk of Bias tool for RCTs and ROBINS-I for observational studies.

**Results:**

Twelve studies (4 RCTs, 8 cohorts) comprising 627 patients and 812 fused levels were included. Single-arm analyses showed high and comparable fusion rates (3D-Ti: 91%, Ti-PEEK: 88%) and similar subsidence (3D-Ti: 19%, Ti-PEEK: 20%) and reoperation rates. Indirect meta-regression found no statistically significant differences between materials for fusion (OR 0.68, 95% CI 0.30–1.50; p = 0.335), subsidence (OR 1.04, 95% CI 0.33–3.22; p = 0.951), reoperation (OR 1.23, 95% CI 0.29–5.24; p = 0.777), or VAS-back (mean difference −0.88, 95% CI -2.49 to 0.73; p = 0.286). Residual heterogeneity was substantial for subsidence (I^2^ = 84%) and VAS-back (I^2^ = 89%), moderate for fusion (I^2^ = 47%), and low for reoperation (I^2^ = 34%). Risk of bias for RCTs was unclear to high (Cochrane RoB), driven primarily by performance bias from unblinded surgeons, and moderate to serious for cohorts (ROBINS-I).

**Conclusion:**

This meta-analysis found no significant differences in radiographic or key clinical outcomes between 3D-Ti and Ti-PEEK cages at intermediate-term follow-up. The evidence suggests clinical equivalence, supporting that cage selection can be guided by factors such as surgeon familiarity, cost, and specific patient imaging needs rather than material properties alone.

## Highlights

• Meta-Analysis comparing 3D-printed Ti vs. Ti-PEEK cages in TLIF (12 studies).
• 627 patients and 812 fused lumbar levels analyzed across 12 studies.
• Pooled fusion rates were 91% for 3D-Ti and 88% for Ti-PEEK.
• Subsidence, reoperation, and VAS-back pain showed no material differences.
• Cage selection can be guided by imaging needs, cost, and surgeon experience.

## 1 Introduction

Low back pain is a common symptom that affects many, with it being the fifth most common reason for visiting a physician in the US, and the lifetime prevalence in US adults being as high as 65-80%.^1^ There are a multitude of causes that could contribute to low back pain, including but not limited to lumbar disc degeneration, spondylosis, spondylolisthesis, facet arthropathy, and spondyloarthropathies.^2^ It is noted that in many patients, non-surgical management, such as physical therapy and nonsteroidal anti-inflammatory drugs, results in the resolution of symptoms.^3^ However, for patients who have persistent, function-limiting symptoms despite conservative treatment, surgical intervention is warranted, where lumbar fusion is the most common surgical treatment.^4^^,^^5^ Transforaminal lumbar interbody fusion (TLIF) is a type of lumbar fusion that has gained popularity due to its safety profile and technical feasibility, with the literature showing that TLIF has lower incidences of nerve injuries and dural tears, as well as shorter operative times.^6^

Regarding TLIF, there are two materials that can be used to make up the interbody cage: titanium (Ti) and polyetheretherketone (PEEK). Ti is the traditional material used and possesses the ability to enhance cell adhesions and osseointegration that provide effective fusion.^7^ However, Ti has a higher modulus of elasticity compared to native bone, leading to increased stress concentration at the bone-implant interface, which could result in a higher risk of cage subsidence and lower clinical outcomes.^8^^,^^9^ PEEK was introduced in the 1990s as an alternative material to Ti, which has a modulus of elasticity that is more similar to cortical bone, thus reducing overall stress on adjacent levels and risk of subsidence. However, in contrast to Ti, the hydrophobic surface of PEEK may restrict its ability to stimulate cell adhesion and osseointegration.^10^ Titanium-coated PEEK (Ti-PEEK) was developed in order to retain both the positive properties of Ti and PEEK.^11^^,^^12^

Although there are studies in the literature that compare PEEK to Ti-PEEK, the authors found that there is limited literature that investigates the differences between Ti and Ti-PEEK. Thus, this meta-analysis aims to bring forth an extensive overview that explores the differences in post-operative outcomes and functional scores between Ti-PEEK and Ti interbody cages in patients who require TLIF.

## 2 Methods

The preferred reporting items for systematic reviews and meta-analyses (PRISMA) guideline was used for this meta-analysis study.^13^

### 2.1 Literature search

A literature search was carried out on PubMed, Embase, and Google Scholar up until April 2025. Search terms related to the procedure (e.g., "Transforaminal Lumbar Interbody Fusion," "TLIF"), device ("Interbody Cage"), materials (e.g., "PEEK," "Titanium," "3D Printed Titanium," "Titanium-Coated PEEK"), and key outcomes ("Fusion," "Subsidence") were combined using Boolean operators. A core search string used was: (Transforaminal Lumbar Interbody Fusion OR TLIF) AND (Interbody Cage) AND (PEEK OR Titanium OR "3D Printed Titanium" OR "Titanium-Coated PEEK") AND (Fusion OR Subsidence). Only studies published in English were included.

### 2.2 Inclusion and exclusion criteria

In order for a study to be included in this study, it must have met the following inclusion criteria: cohort study, case control study, or randomized controlled trial (RCT), comparison groups consisted of 3D-Ti and/or Ti-PEEK, noted at least one outcome of interest, and comparing to either Titanium or PEEK cage. Outcomes of interest included fusion rate, cage subsidence rate, re-operation rate, and visual-analogue scores (VAS), including VAS-back and VAS-leg. The minimum follow-up time was 6 months. Studies that did not adhere to the described inclusion criteria and/or were systematic reviews, meta-analyses, case reports, biomechanical studies, conference abstracts, non-English studies, or where full texts were not accessible were excluded from this study. All authors independently screened the initial studies gathered from the literature search and applied the inclusion/exclusion criteria. Unfortunately, the authors found that there are a limited number of studies that directly compare Ti to Ti-PEEK. To account for this, the authors instead included comparison studies that investigated Ti or Ti-PEEK and carried out an indirect comparison.

### 2.3 Assessment of study quality

For RCTs, study quality was assessed using the Cochrane Risk of Bias tool,^14^ and for case-control and cohort studies, ROBINS-I was used.^15^ All of the reviewers completed the analysis independently and reconciled any disagreements through discussion and consultation with a third author when needed. Each article was labeled as a low risk, unclear risk, or high risk of bias based on the following parameters: random sequence generation (selection bias), allocation concealment (selection bias), blinding of participants and personnel (performance bias), blinding of outcome assessment (detection bias), incomplete outcome data (attrition bias), selective reporting (reporting bias), and other bias.

For all non-randomized cohort and case-control studies, we applied the Risk Of Bias In Non-randomized Studies-of Interventions (ROBINS-I) tool. ROBINS-I evaluates seven bias domains: (1) confounding, (2) classification of interventions, (3) selection of participants into the study or analysis, (4) deviations from intended interventions, (5) missing data, (6) measurement of outcomes, and (7) selection of the reported result. Within each domain, responses to the signaling questions were given one of four ratings: Low risk (except for uncontrolled confounding in Domain 1), Moderate, Serious, or Critical. Following ROBINS-I guidance, the overall study judgement corresponded to the highest risk level recorded in any domain.

### 2.4 Data extraction

Two reviewers independently extracted data into a piloted spreadsheet, resolving disagreements by consensus with third-reviewer adjudication as needed. We captured study characteristics (first author, year, country, design), TLIF approach (open, minimally invasive, or mixed), follow-up duration, and arm allocation by cage material: 3D-printed porous titanium (3D-Ti) vs titanium–polyetheretherketone (Ti-PEEK). For each arm, we recorded the unit and size of analysis (patients and, when available, operated levels) and available baseline characteristics. When multiple postoperative assessments were reported, we prioritized the longest follow-up and noted its timing.

Dichotomous outcomes (fusion, subsidence, reoperation) were recorded as events and denominators, following each study's definition of subsidence. Continuous outcomes (Oswestry Disability Index [ODI], Visual Analog Scale [VAS] pain, back and/or leg) were extracted as means, standard deviations (SDs), and sample sizes; VAS was harmonized to a 0–10 scale and ODI to 0–100. When outcomes were reported per-patient and per-level, per-patient data were used for primary analyses (per-level for sensitivity). SDs were derived from CIs, SEs, or P-values when necessary; medians with IQRs/ranges were converted using established methods, and nonconvertible data were excluded from meta-analysis for that endpoint. For multi-arm or stratified reports, subgroups were combined per Cochrane guidance or kept separate if fully independent.

### 2.5 Statistical analysis

All statistical computations were carried out in Review Manager (RevMan, version 5.4; The Cochrane Collaboration) supplemented by R (v 4.3.2). Study-level data were imported from the master worksheet created from the data extracted from the studies, and analyzed on a per-segment basis whenever both segment and patient counts were reported.

For each outcome we first performed separate single-arm meta-analyses for titanium (Ti) and titanium-coated PEEK (Ti-PEEK) cages. Radiographic fusion, cage subsidence and re-operation were treated as proportions; these were logit-transformed to stabilize variances (PLO metric), with a continuity correction of 0.5 added to studies reporting zero events. Visual-analogue pain scores (VAS-back and VAS-leg) and Oswestry Disability Index values were pooled as raw means, and any VAS values originally presented on a 0–100 scale were divided by ten to harmonize all studies to a 0–10 scale. Each meta-analysis was fitted with a random-effects model using restricted-maximum-likelihood (REML) estimation, and studies were weighted by the inverse of their within-study variance. Between-study heterogeneity was summarized with τ^2^, Cochran's Q and the I^2^ statistic. Forest plots display individual study estimates, inverse-variance weights and the REML-pooled effect. Pooled proportions were back-transformed to the original scale for interpretation.

To compare materials indirectly, the Ti and Ti-PEEK datasets were stacked and a random-effects meta-regression was run with material (Ti-PEEK = 1, Ti = 0) as a moderator. For dichotomous outcomes this produced a logit difference that was exponentiated to yield the ratio of pooled proportions (Ti-PEEK/Ti) with 95% confidence intervals, while for continuous outcomes it yielded an absolute mean difference (Ti-PEEK minus Ti). Two-sided p-values <0.05 were considered statistically significant.

## 3 Results

### 3.1 Study selection

**Fig. 1:**
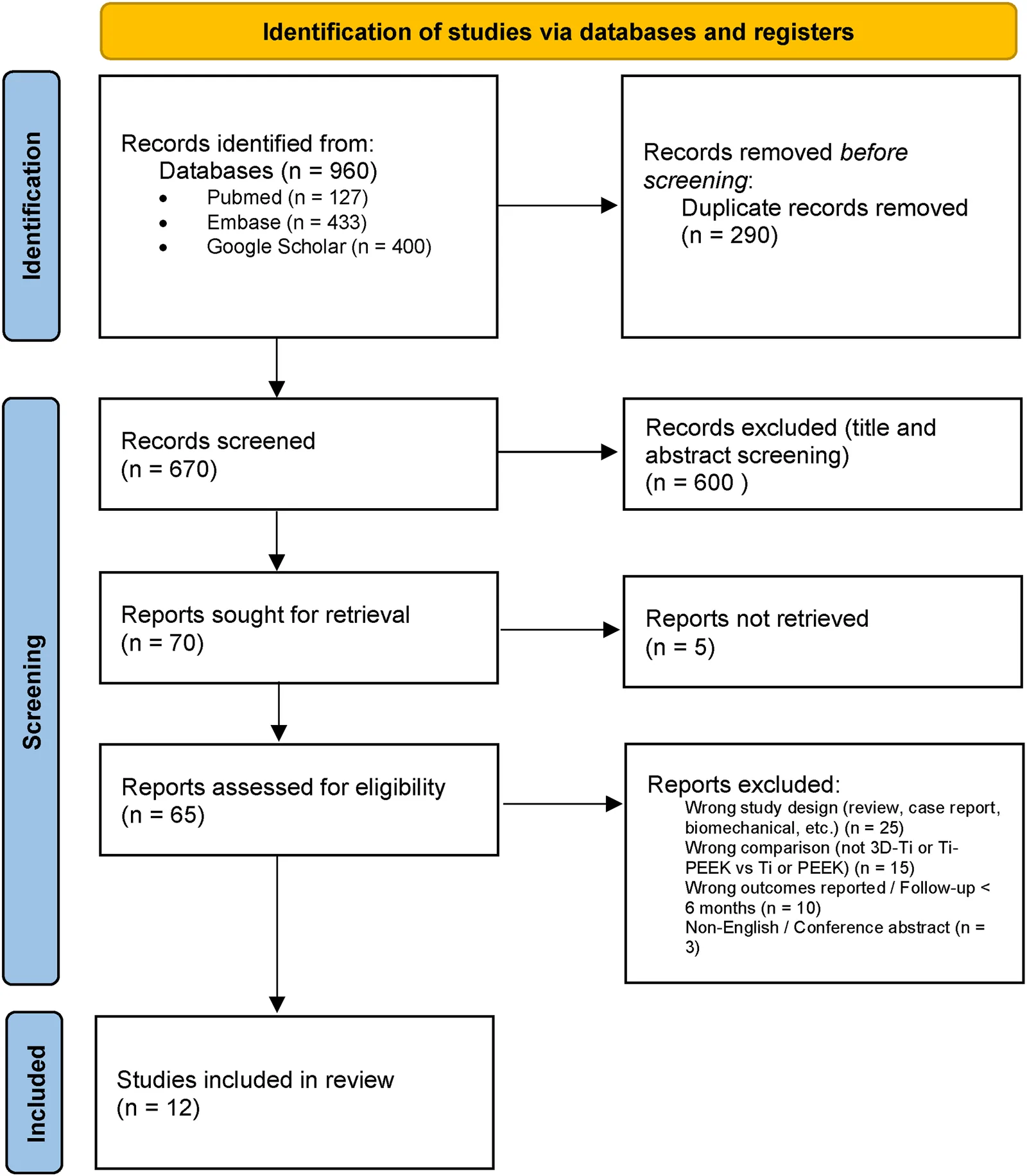
PRISMA 2020 flow diagram of study identification, screening, and inclusion. From 960 records identified across PubMed (n = 127), Embase (n = 433), and Google Scholar (n = 400), 290 duplicates were removed, and 670 titles and abstracts were screened. After excluding 600 records, 70 reports were sought for retrieval, and 5 were unobtainable. Of 65 full-text reports assessed for eligibility, 53 were excluded (25 for the wrong study design, 15 for the wrong comparison, 10 for the wrong outcomes or follow-up < 6 months, 3 for non-English or conference abstracts), yielding 12 studies included in the qualitative and quantitative syntheses.

The database search identified 960 records (PubMed, n = 127; Embase, n = 433; Google Scholar, n = 400). After removal of 290 duplicates, 670 titles and abstracts were screened; 600 records were excluded. Seventy reports were sought for retrieval, of which five were not retrieved. Sixty-five full texts were assessed for eligibility; 53 were excluded (25 wrong study design, 15 wrong comparison, 10 wrong outcomes or follow-up < 6 months, 3 non-English or conference abstracts). Twelve studies^11^^,^^16^, ^17^, ^18^, ^19^, ^20^, ^21^, ^22^, ^23^, ^24^, ^25^, ^26^ met inclusion criteria and were included in the qualitative and quantitative syntheses (Fig. 1).

### 3.2 Study characteristics

Across the 12 studies, there were 627 patients and 812 fused levels overall. Of these levels, 477 received 3D-printed porous titanium (3D-Ti) cages and 335 received titanium-coated PEEK (Ti-PEEK) cages. Designs included four randomized controlled trials and eight observational cohorts. Most studies reported outcomes at 12 months, with single- or two-level TLIF being most common. Detailed study characteristics are presented in Table 1.

**Table 1:** Characteristics of included studies.

Study	Country	Design	Approach	F/U (mo)	Comparison	3D-Ti arm	Ti-PEEK arm	Note
Chahlavi 2024	USA	Retrospective, self-controlled	Open	12	3D-Ti vs Ti-PEEK	48 patients; 56 levels	48 patients; 52 levels	a
Jacob 2025	USA	Retrospective cohort	Open/Mixed	12	3D-Ti vs Ti-PEEK	140 patients; 202 levels	60 patients; 75 levels	b
Kim 2022	South Korea	Retrospective cohort	MIS	12	3D-Ti vs PEEK	40 patients; 40 levels	—	
Masamoto 2025	Japan	Retrospective cohort	Open	12	Ti-PEEK vs PEEK	—	30 patients; 36 levels	
Rickert 2017	Germany	RCT (pilot)	Open	12	Ti-PEEK vs PEEK	—	20 patients; 24 levels	c
Singhatanadgige 2022	Thailand	RCT	MIS	12	Ti-PEEK vs PEEK	—	41 patients; 49 levels	
Sultana 2023	South Korea	Retrospective cohort	MIS	20	3D-Ti vs PEEK	70 patients; 90 levels	—	
Toop 2023	USA	RCT	Open	6	3D-Ti vs PEEK	24 patients; 34 levels	—	
Vanek 2024	Czech Republic	RCT	Open	24	Ti-PEEK vs PEEK	—	40 patients; 40 levels	
Yao 2023	Taiwan	Retrospective case-control	MIS	24	Ti-PEEK vs PEEK	—	27 patients; 27 levels	
You 2024 (Large 3D-Ti)	South Korea	Retrospective cohort	BE-TLIF (endoscopic)	12	3D-Ti vs PEEK	24 patients; 24 levels	—	d
You 2024 (Regular 3D-Ti)	South Korea	Retrospective cohort	BE-TLIF (endoscopic)	12	3D-Ti vs PEEK	31 patients; 31 levels	—	d
Zhu 2021	China	Prospective cohort	Open	29.7	Ti-PEEK vs PEEK	—	32 patients; 32 levels	

BE-TLIF, biportal endoscopic transforaminal lumbar interbody fusion; F/U, follow-up; HA, hydroxyapatite; MIS, minimally invasive surgery; mo, months; PEEK, polyetheretherketone; RCT, randomized controlled trial; Ti, titanium; Ti-PEEK, titanium-coated PEEK; 3D-Ti, three-dimensionally printed porous titanium.

(a) Self-controlled design: each of 48 patients contributed both a 3D-Ti and a Ti-PEEK level. Subsidence and fusion assessed on a subset of levels with imaging follow-up; reoperation tracked per-patient.

(b) Subsidence and fusion analyzed per-level among levels with ≥1-year imaging follow-up; ODI standard deviation not reported and therefore not pooled.

(c) Subsidence and fusion reported as percent vertebral-body height loss only; per-level event counts not extractable, so Rickert was excluded from the subsidence and fusion proportion meta-analyses (included for VAS, ODI, and reoperation).

(d) You 2024 contributed two cage configurations (Large 3D-Ti and Regular 3D-Ti), treated as separate arms in the meta-analyses.

### 3.3 Pooled outcomes

**Fig. 2:**
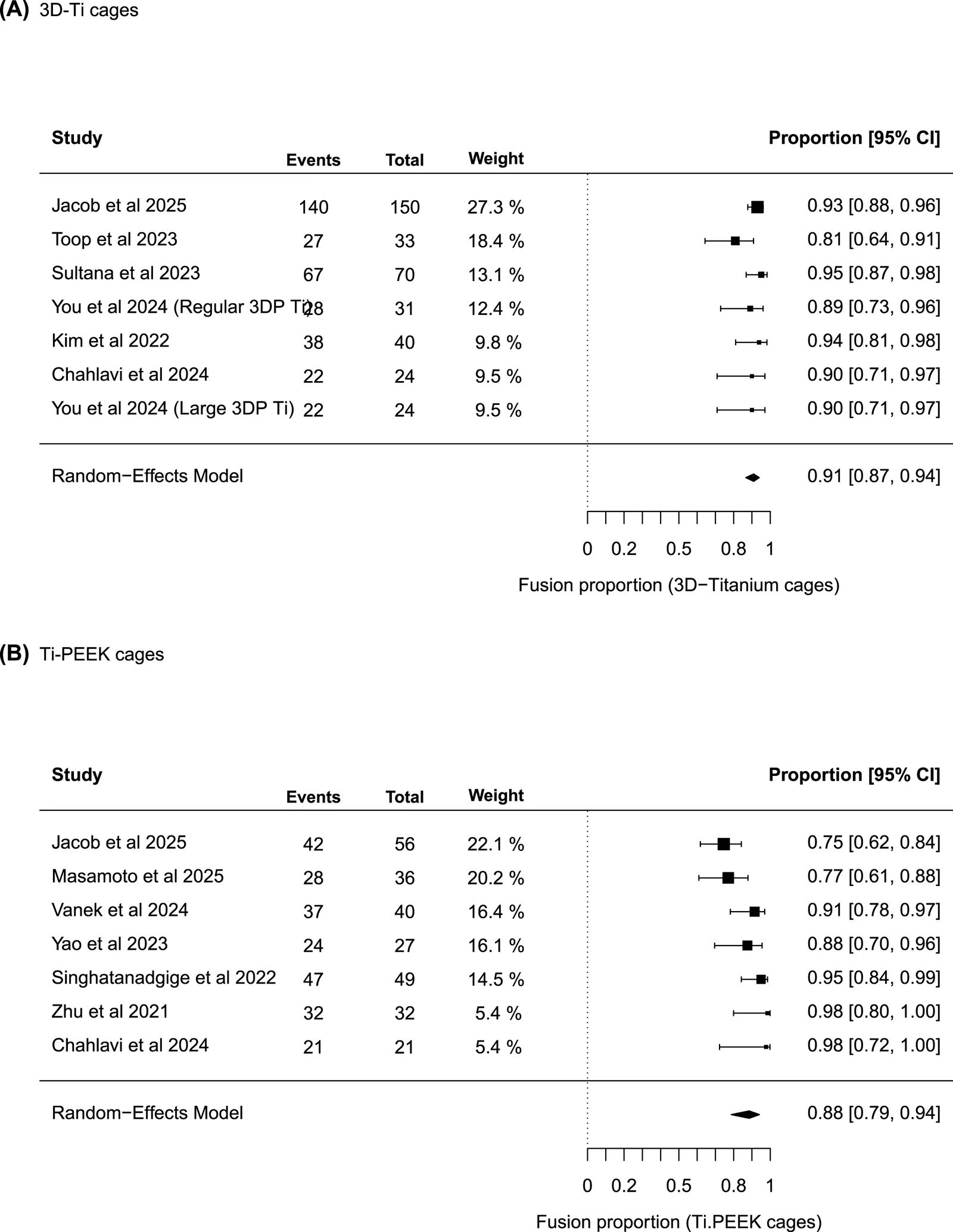
Forest plots of pooled radiographic fusion proportions for (A) 3D-printed porous titanium (3D-Ti) cages and (B) titanium-coated polyetheretherketone (Ti-PEEK) cages. Each row presents study-level event counts, denominators, inverse-variance weights, and proportions with 95% confidence intervals. The summary diamond represents the random-effects pooled estimate from a logit-transformed (PLO) model fitted by restricted maximum likelihood. Pooled fusion proportion was 91% (95% CI 87–94) for 3D-Ti and 88% (79–94) for Ti-PEEK.

**Fig. 3:**
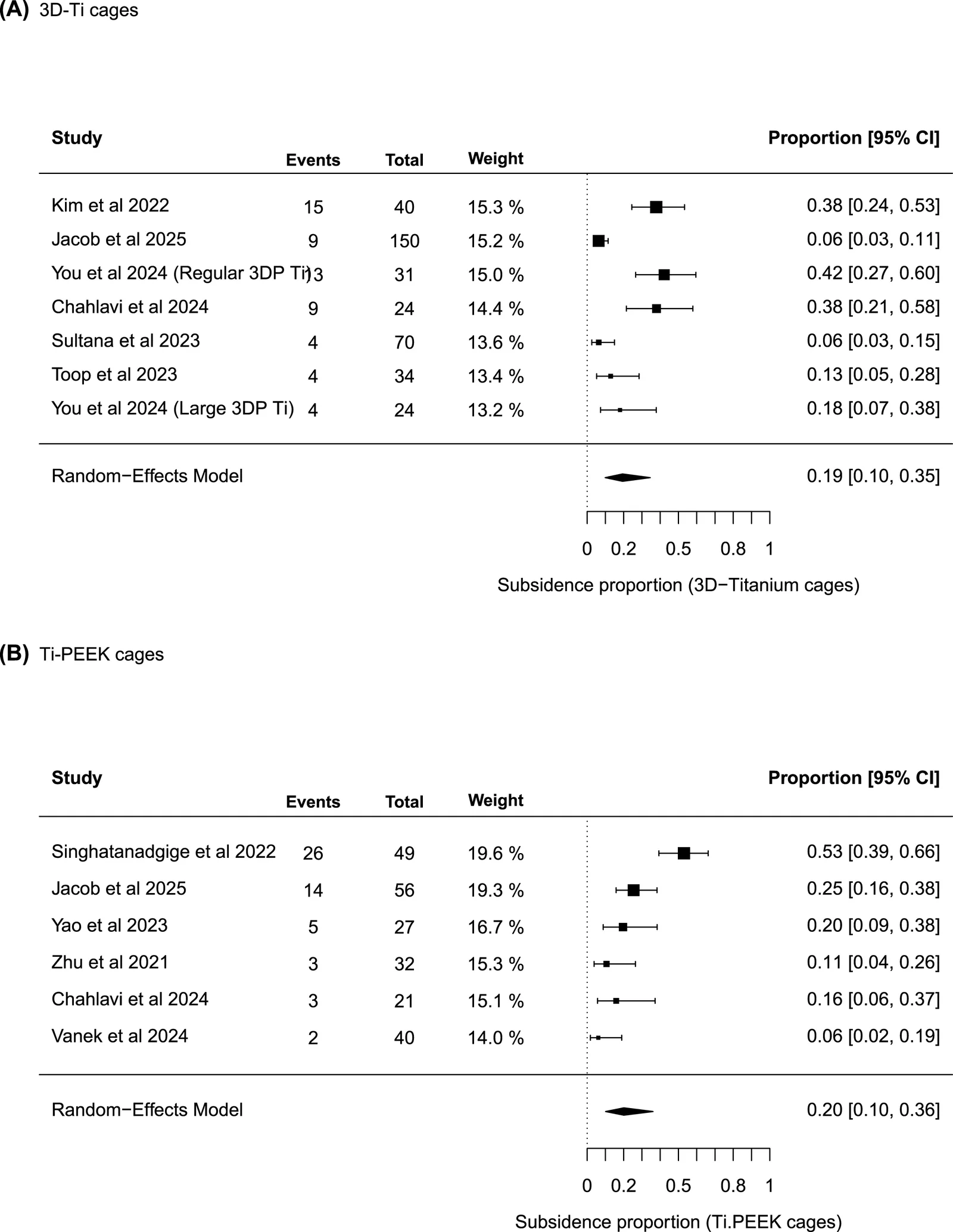
Forest plots of pooled cage subsidence proportions for (A) 3D-Ti cages and (B) Ti-PEEK cages. Random-effects model with REML estimation. Pooled subsidence proportion was 19% (95% CI 10–35) for 3D-Ti and 20% (10–36) for Ti-PEEK. Substantial between-study heterogeneity was observed (I^2^ = 84%), reflecting differences in subsidence definitions, imaging modality, and follow-up across studies. Rickert et al. (2017) were excluded from this analysis because subsidence was reported only as percent vertebral-body height loss, without extractable per-level event counts.

**Fig. 4:**
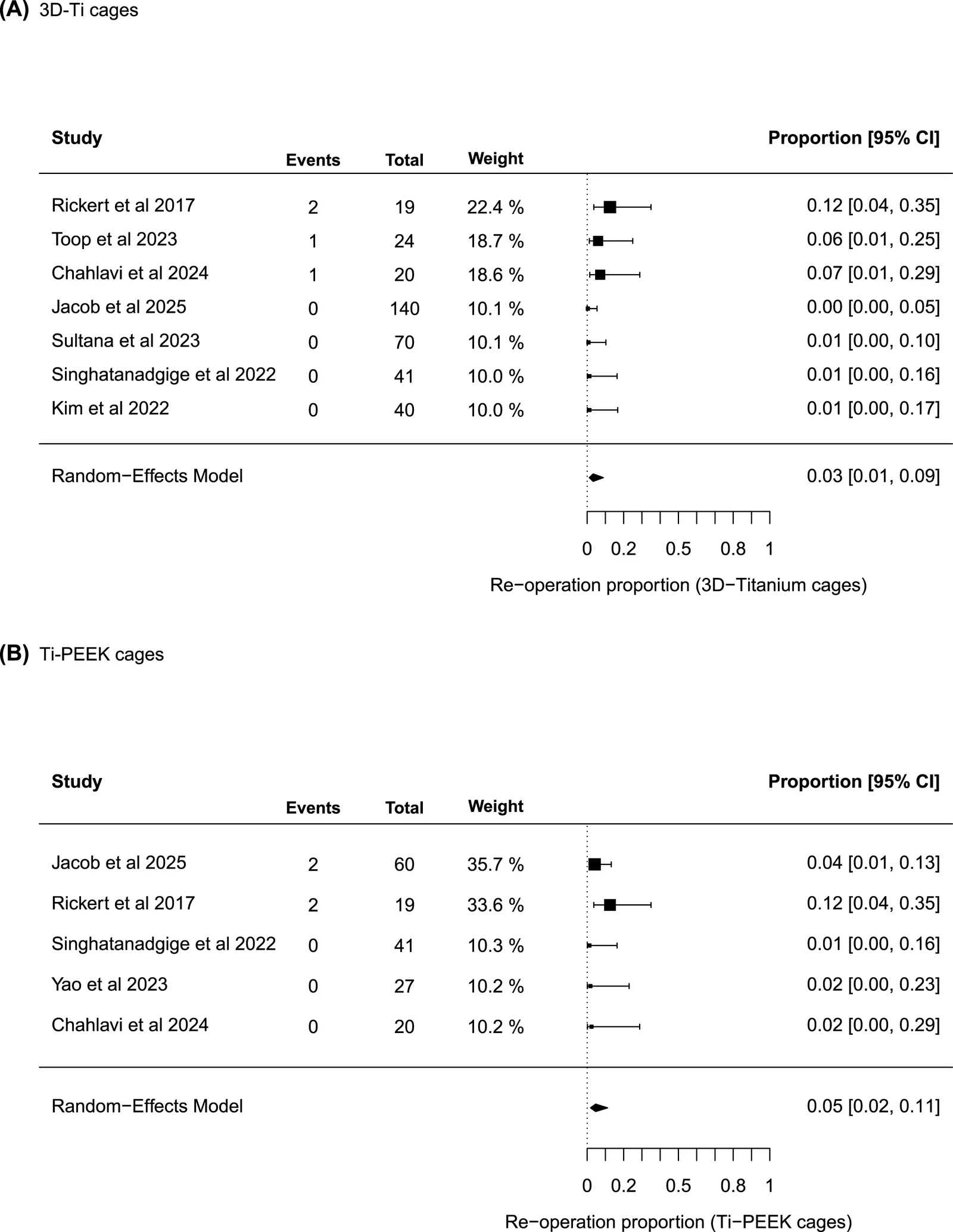
Forest plots of pooled reoperation proportions for (A) 3D-Ti cages and (B) Ti-PEEK cages. Random-effects model with REML estimation; a continuity correction of 0.5 was added to studies reporting zero events. Pooled reoperation proportion was 3.3% (95% CI 1.2–8.8) for 3D-Ti and 4.6% (1.9–11.1) for Ti-PEEK.

**Fig. 5:**
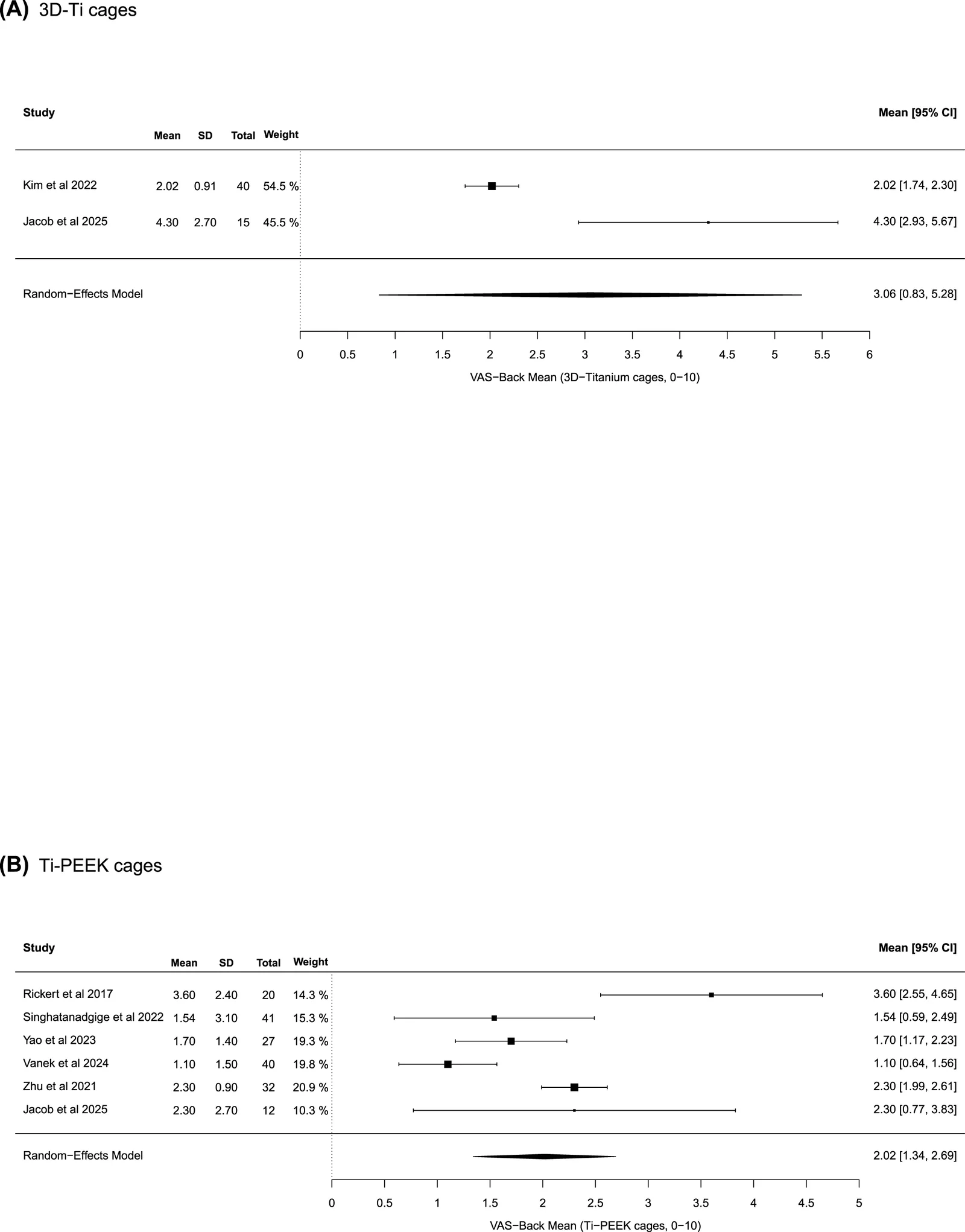
Forest plots of pooled mean Visual Analog Scale back-pain (VAS-back) scores at final follow-up for (A) 3D-Ti cages and (B) Ti-PEEK cages, harmonized to a 0–10 scale. Random-effects model with REML estimation, weighted by inverse within-study variance. Pooled mean VAS-back was 3.06 (95% CI 0.83–5.28) for 3D-Ti and 2.02 (1.34–2.69) for Ti-PEEK.

Single-arm random-effects syntheses showed high and similar final fusion rates for both materials (3D-Ti: 91% [95% CI 87–94]; Ti-PEEK: 88% [79–94]; Fig. 2). Subsidence proportions were comparable (3D-Ti: 19% [95% CI 10–35]; Ti-PEEK: 20% [10–36]; Fig. 3). Reoperation rates were low for both groups (3D-Ti: 3.3% [95% CI 1.2–8.8]; Ti-PEEK: 4.6% [1.9–11.1]; Fig. 4). Mean VAS-back at final follow-up was 3.06 [95% CI 0.83–5.28] for 3D-Ti and 2.02 [1.34–2.69] for Ti-PEEK (Fig. 5). VAS-leg and ODI were not synthesized due to insufficient harmonizable data.

### 3.4 Indirect comparative meta-regression

Between-material meta-regression detected no statistically significant differences for any endpoint. Using a coding where OR > 1 indicates higher odds with Ti-PEEK (and OR < 1 favors 3D-Ti), the models yielded: fusion OR 0.68 (95% CI 0.30–1.50; p = 0.335), subsidence OR 1.04 (0.33–3.22; p = 0.951), and reoperation OR 1.23 (0.29–5.24; p = 0.777). For VAS-back, the mean difference was −0.88 (−2.49 to 0.73; p = 0.286), favoring Ti-PEEK but not significantly. Residual between-study heterogeneity not explained by material was substantial for subsidence (I^2^ = 83.6%; τ^2^ = 0.875; Q(11) = 73.6, p < 0.001) and VAS-back (I^2^ = 88.8%; τ^2^ = 0.817; Q(6) = 39.2, p < 0.001), moderate for fusion (I^2^ = 47.1%; τ^2^ = 0.249; Q(12) = 22.8, p = 0.029), and low for reoperation (I^2^ = 33.9%; τ^2^ = 0.525; Q(10) = 14.3, p = 0.161). Material accounted for a small fraction of fusion heterogeneity (R^2^ = 16.0%) but none of the variability in subsidence, reoperation, or VAS-back.

### 3.5 Risk of bias

Risk-of-bias assessments mirrored study design. Among the four RCTs (Cochrane RoB), two were judged at high overall risk of bias and two at unclear overall risk. All four trials shared high risk for performance bias due to the inability to blind operating surgeons, an unavoidable limitation of surgical-device trials. The two high-risk trials had additional concerns: one with high risk for detection bias from unblinded outcome assessment, and one with high risk for other bias due to early stopping after a planned interim analysis with industry funding. Among the eight cohorts (ROBINS-I), five were judged at overall serious risk and three at overall moderate risk. Serious-risk ratings most commonly reflected confounding, post-baseline exclusions, and/or missing data. Full, study-level domain ratings are provided in Table 2.

**Table 2:**
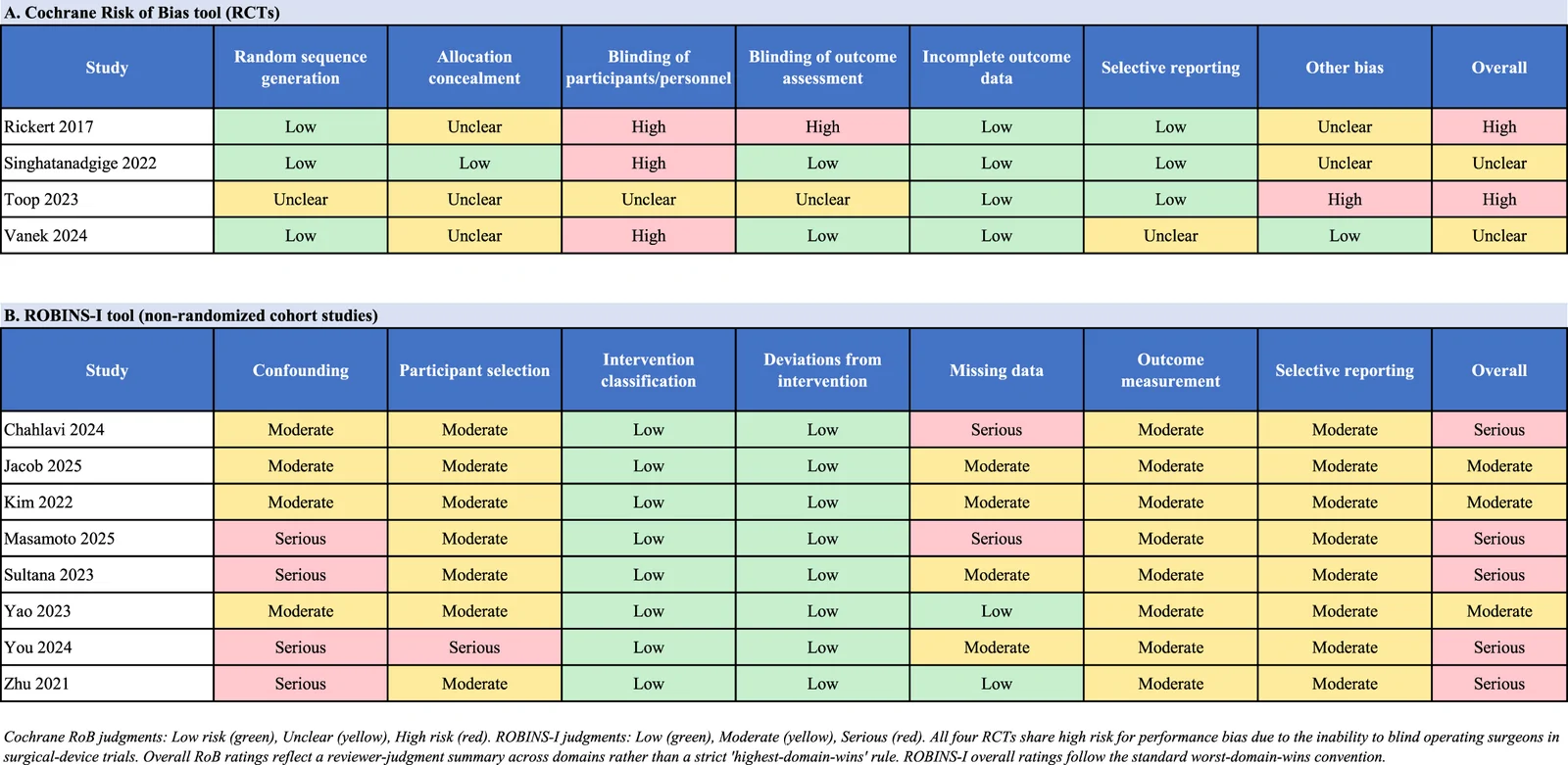
Risk of bias assessments for randomized controlled trials (Cochrane Risk of Bias tool) and non-randomized cohort studies (ROBINS-I).

## 4 Discussion

### 4.1 Principal findings

In this meta-analysis of 12 studies encompassing 627 patients and 812 fused levels (477 3D-printed porous titanium (3D-Ti) and 335 titanium-coated PEEK (Ti-PEEK)), we found no statistically significant differences between cage materials in final fusion, subsidence, reoperation, or VAS-back at the longest available follow-up. These results persisted in random-effects single-arm syntheses and in indirect comparative meta-regression. Together, the data suggest that while advanced surface technologies may influence the tempo of early healing, they do not translate into clear superiority of either material in mature radiographic or clinical outcomes after TLIF.

### 4.2 Context within the literature

Biomechanical and surface-biology rationales predict divergent performance: 3D-Ti offers a highly porous, osteoconductive lattice that promotes on- and in-growth, whereas Ti-PEEK seeks to retain PEEK's bone-like modulus and radiolucency while adding a titanium surface for osseointegration. Our synthesis indicates that these theoretical advantages largely equilibrate at 12–24 months, aligning with contemporary overviews of cage evolution and material properties.

Several influential primary studies illustrate why pooled effects converge. Two randomized trials observed a marked advantage for titanium-based surfaces at early time points (e.g., 3–6 months) that disappeared by one year, with no differences in patient-reported outcomes, consistent with an acceleration of fusion rather than a higher final success rate.^11^^,^^22^ Conversely, longer-term randomized evidence has shown lower subsidence with Ti-PEEK yet no difference in final fusion or symptoms, balancing signals that might otherwise favor 3D-Ti. Finally, large retrospective cohorts reporting strong advantages for 3D-Ti were limited by important confounding (e.g., differential use of BMP) and design differences (e.g., cage geometry), tempering their influence on clinical decision-making.

### 4.3 Clinical implications

For most TLIF indications, the present evidence supports selecting either 3D-Ti or Ti-PEEK without expectation of materially different long-term fusion or symptom relief. Practical considerations may therefore guide choice. Ti-PEEK preserves radiolucency, potentially facilitating adjudication of fusion on plain radiographs and CT; 3D-Ti provides aggressive surface topography and initial grip but is radiopaque. Cost, surgeon familiarity, and imaging needs should be weighed alongside patient-specific factors (bone quality, level, need for early radiographic assurance). Moreover, several studies highlight that variables beyond material likely exert larger effects on subsidence and early radiographic appearance than material alone. These include cage footprint/size, endplate preparation, and surgical approach (open, MIS, or endoscopic).

### 4.4 Strengths and limitations

Strengths of this work include protocolized extraction, risk-of-bias assessment tailored to design, and prespecified harmonization of scales and units. Importantly, four randomized trials contribute higher-quality evidence; however, the literature remains heterogenous in imaging modality (CT vs radiographs), follow-up timing, cage geometry, graft/biologic use, and unit of analysis (per-patient vs per-level). The predominance of indirect comparisons required meta-regression rather than head-to-head pooling, and two patient-reported outcomes (VAS-leg and ODI) could not be synthesized due to sparse data. Observational evidence suggesting material differences frequently coincided with confounding (e.g., BMP use, historical cohorts) that likely inflated effect sizes. Collectively, these limitations argue for cautious interpretation of small between-material differences and for emphasizing consistent, patient-centered outcomes. Most included studies did not stratify outcomes by sex or gender, and we were therefore unable to assess whether material performance differs between male and female patients; future studies should report sex-disaggregated outcomes to address this gap.

## 5 Conclusion

Across 12 studies (627 patients; 812 levels), 3D-printed porous titanium and titanium-coated PEEK cages show no material differences in final fusion, subsidence, reoperation, or VAS-back at 12–24 months. Given that the randomized trials were predominantly limited by unavoidable performance bias inherent to surgical-device trials, while many observational cohorts carried additional risk from confounding and missing data, the most credible evidence supports clinical equivalence; therefore, cage selection should be guided by imaging needs, cost/availability, surgeon familiarity, and patient-specific factors rather than material alone. Well-designed, head-to-head RCTs with matched geometry, standardized biologics, CT-based fusion grading, and ≥24-month follow-up remain a priority.

## Guardian Patient's consent

NA.

## Ethics approval and consent to participate

Not applicable. This article is a systematic review of previously published studies.

## Consent for publication

Not applicable.

## Availability of data and materials

All data generated or analyzed during this study are included in this published article.

## Ethical statement

We confirm that all authors have read and approved the manuscript, and no other individuals meet the authorship criteria but are not listed. We have also agreed upon the order of authorship as presented. Furthermore, we assure you that this manuscript has not been submitted elsewhere.

Thank you for considering our work for publication. We deeply appreciate the time and effort you and the journal's esteemed review team will invest in our manuscript. Please feel free to reach out if you have any questions or require additional information.

## Credit author statement

YY, TC, and JL contributed to the conception and design of the study, performed the literature search and data extraction, conducted the data analysis, drafted the initial manuscript, and revised the manuscript. JL supervised the study. All authors read and approved the final version of the manuscript and agree to be accountable for all aspects of the work.

## Funding information

This research was conducted independently and did not receive specific funding from public, commercial, or not-for-profit agencies.

## Competing interests

The authors declare that they have no competing interests.
